# Meta-analysis of transcriptomic datasets identifies genes enriched in the mammalian circadian pacemaker

**DOI:** 10.1093/nar/gkx714

**Published:** 2017-08-18

**Authors:** Laurence A. Brown, John Williams, Lewis Taylor, Ross J. Thomson, Patrick M. Nolan, Russell G. Foster, Stuart N. Peirson

**Affiliations:** 1 Sleep and Circadian Neuroscience Institute (SCNi), Nuffield Department of Clinical Neurosciences, University of Oxford, Oxford, OX1 3RE, UK; 2 MRC Harwell Institute, Harwell Campus, Oxfordshire OX11 0RD, UK

## Abstract

The master circadian pacemaker in mammals is located in the suprachiasmatic nuclei (SCN) which regulate physiology and behaviour, as well as coordinating peripheral clocks throughout the body. Investigating the function of the SCN has often focused on the identification of rhythmically expressed genes. However, not all genes critical for SCN function are rhythmically expressed. An alternative strategy is to characterize those genes that are selectively enriched in the SCN. Here, we examined the transcriptome of the SCN and whole brain (WB) of mice using meta-analysis of publicly deposited data across a range of microarray platforms and RNA-Seq data. A total of 79 microarrays were used (24 SCN and 55 WB samples, 4 different microarray platforms), alongside 17 RNA-Seq data files (7 SCN and 10 WB). 31 684 MGI gene symbols had data for at least one platform. Meta-analysis using a random effects model for weighting individual effect sizes (derived from differential expression between relevant SCN and WB samples) reliably detected known SCN markers. SCN-enriched transcripts identified in this study provide novel insights into SCN function, including identifying genes which may play key roles in SCN physiology or provide SCN-specific drivers.

## INTRODUCTION

Life on Earth has evolved under a predictably changing cycle of light and darkness and, as a result, virtually all organisms demonstrate striking changes in physiology and behaviour over the 24-h day. These rhythms are not simply a response to the changing environment, but persist under constant conditions—providing evidence for the existence of an endogenous biological clock. In mammals, the site of the master circadian pacemaker is the suprachiasmatic nuclei (SCN) in the anterior hypothalamus (1). The SCN receives light information from the retina via the retinohypothalamic tract, synchronizing (entraining) SCN rhythms to the external environment. SCN lesions result in loss of physiological and behavioural rhythms, and transplantation of foetal SCN can restore rhythmicity with a period consistent with that of the donor tissue (2). The circadian clock is the product of an intracellular transcriptional-translational feedback loop (TTFL), comprised of a number of so-called ‘clock genes’. Whilst clock genes are expressed in tissues throughout the body, the coordinated function of the circadian system depends upon neural, hormonal and behavioural output of the SCN pacemaker (3–5).

The identification of mammalian clock genes has critically depended upon the application of forward and reverse genetics. Large scale mutagenesis projects have been invaluable in identifying novel mouse mutants with circadian phenotypes. Mapping the underlying mutation has led to the identification of a number of key clock genes, including *Clock* (6), *Fbxl3* (7), *Fbxl21* (8) and *Zfhx3* (9). By contrast, reverse genetics has been used to identify homologs of *Drosophila* clock genes (identified by forward genetics), including *Period 1–3* (10–12) and *Cryptochrome 1–2* (13,14) as well as proteins that interact with known clock components, such as *Arntl* (*Bmal1*, (15)). A key feature of many of the core clock genes is that they are rhythmically expressed over the 24-h cycle. Therefore, studies to identify new clock genes have often taken the approach of identifying transcripts that are rhythmically expressed in the SCN or other peripheral clocks, under either entrained light/dark conditions or over one or more circadian cycles under constant conditions (16). These studies have been successful in identifying novel clock genes as well as genes important for SCN function (17,18). However, not all clock genes are rhythmically expressed, and rather than changing in abundance, some components of the TTFL may show rhythmic post-translational modification or simply depend upon interactions with other rhythmic components. Moreover, many genes involved in key functions of the SCN are not elements of the TTFL. For example, a number of neuropeptides and their receptors play key roles in SCN physiology (such as vasoactive intestinal polypeptide, VIP (19) and VIPR2 (20)), as do GABA receptors (21).

An alternative strategy to identify mechanisms critical for SCN function is to characterize those genes that are selectively enriched in the SCN. The most straightforward way of identifying SCN-enriched genes is to directly compare the transcriptome of SCN against the whole brain. Despite the value of this approach, only a few such studies exist - focusing on multiple brain regions (22), or specifically on a subset of genes (23). Another advantage of this approach is that it enables SCN-specific genes to be identified, providing critical tools for conditional transgenesis. As many clock genes are expressed throughout the body, studies of constitutive knockout mouse models can be problematic due to developmental effects or differing roles of these genes in other target tissues/organs. As a result, many researchers are looking to the use of conditional knockouts, for example, using Cre-lox technology (24,25). The identification of genes that are specifically and highly enriched in the SCN may not only reveal new biological insights regarding SCN physiology, but also has the potential to produce SCN-specific drivers, which would be of benefit to the circadian community.

Studies using transcriptomic approaches face several well-characterized challenges (26). Biological and technical variance result in a degree of noise in any study, but when making comparisons between thousands of genes, false positive rates become a major problem. Using *P* < 0.05, comparing the expression of ∼20 000 transcripts would be expected to give 1000 false positives—genes that would appear significant even though they are unchanged. As such, false discovery rates are corrected to account for this issue. However, due to the cost of running transcriptomic experiments, sample sizes are often limited. This results in reduced power—that is the ability to identify real differences where they exist, which can result in biologically relevant findings being missed. As such, transcriptomic studies are often a statistical balancing act—resulting in a list of candidate genes which may contain false positives, and may be missing real genes of interest. The downstream effects of this compromise will often be that examining pathways or gene ontologies may be uninformative or misleading.

One way of addressing these statistical issues is to gather as much relevant data as possible from all available sources. Such approaches are widely applied to clinical studies in the form of meta-analysis—a combination of all available data in the literature to enable better outcome decisions, which are now considered essential in clinical science (27). Published microarray data are widely available via deposition into public databases, including NCBI’s Gene Expression Omnibus (28) and EBI’s ArrayExpress (29). As such, meta-analysis of microarray data is a topic of growing interest. This has led to a series of possible methods for dealing with the problems inherent in comparing different microarray platforms with varying numbers of probes for different numbers of transcripts. The methods for drawing together a series of experiments with differing biological and technical variance are numerous, and depend partly on the quality and detail of the available data. Where only lists of significant genes are available, methods such as vote-counting provide the most straightforward approach to meta-analysis (30). Venn-diagrams are often used as a simple form of vote counting, focusing on genes that pass both a fold-change criteria and one or more statistical tests. A problem of this approach is that the pre-processing methods and inclusion criteria used by the original authors to construct each gene list are unlikely to be comparable across all data sources (16). Alternative approaches make use of original raw data by combining either rank-orders of genes (31) or *P*-values as measures of significance (32). Finally, weighting the contribution of each data point for a gene by the inverse of the variance of that data provides a way of comparing data across studies, assuming that lower variance is an indicator of reproducibility (33). These methods are discussed in detail by Ramasamy *et al.* (34), which provides the basis for our analysis.

**Figure 1.**
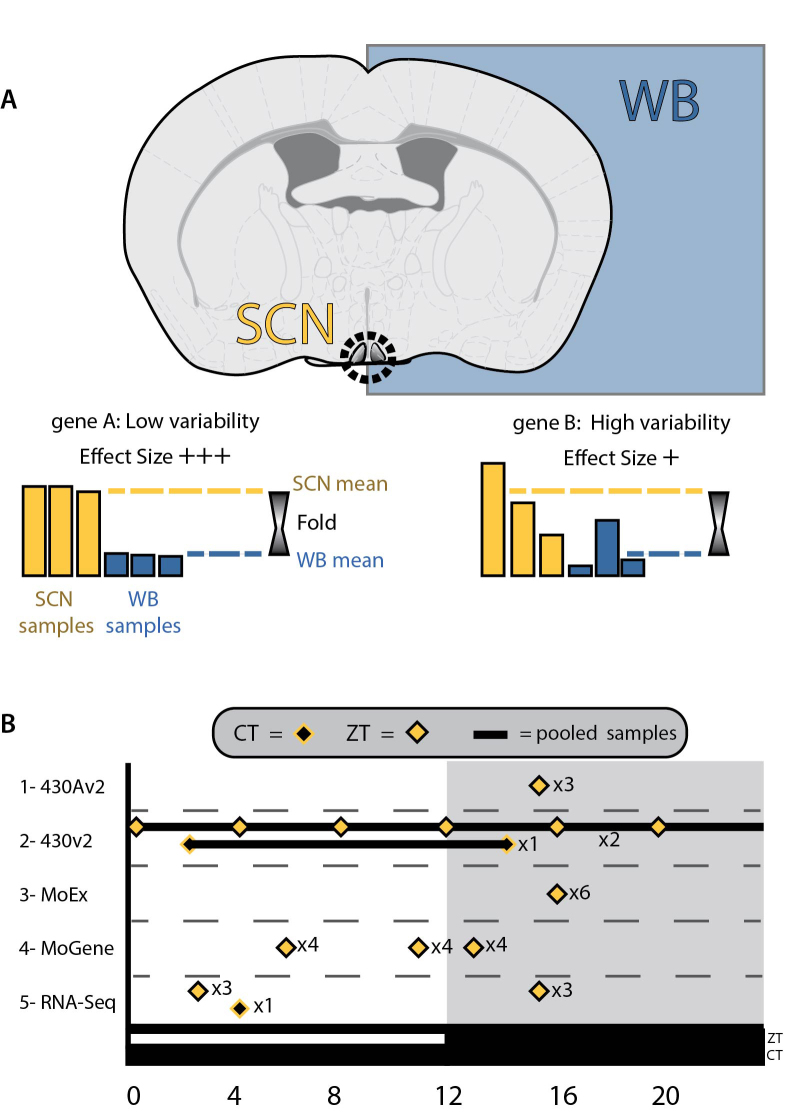
(**A**) The rationale for meta-analyses, with differences in expression between SCN and whole brain samples used to calculate effect sizes for each gene and transcriptomic platform. Treating each platform as an individual study allows data from disparate sources to be brought together in a meta-analysis. (**B**) The times at which SCN samples were collected, showing a range of protocols, including collection by external/zeitgeber times (ZTs) and internal/circadian times(CTs), as well as individual samples at multiple time points and the pooling of RNA samples prior to measurements of transcripts.

Here, we describe a meta-analysis of expression data from SCN and whole brain across a range of microarray technologies and incorporating recent RNA-Seq data (Figure 1). We use the term ‘platform’ in the rest of the paper to refer to each different type of microarray technology (e.g. Mouse Exon ST1.0 arrays) or RNA-Sequencing. This meta-analysis enables us to reliably identify transcripts enriched in the SCN. These transcripts provide novel insights into the function of the master circadian pacemaker, providing new candidates that may play key roles in SCN function, as well as providing new targets for SCN-specific gene targeting.

## MATERIALS AND METHODS

### Data-mining to identify SCN and WB datasets for Affymetrix platforms

Analysis of publicly available datasets revealed that a large part of the apparent variation in any comparison was attributable to differences in the different array platforms. For this reason samples from a particular microarray platform could only be used if both SCN and whole brain (WB) data were available for that platform. In addition, investigations were limited to the Affymetrix microarray platforms (by far the most prominent platforms with suitable data) and we only used data where the original array scan files (.CEL files) were available. This has previously been referred to as feature level extraction output (FLEO), and allows a higher level of control over the processing of array files and consequently the accuracy of comparisons at the gene level (34).

Datasets were identified using simple searches of depositories of publicly available microarray data: NCBI’s Gene Expression Omnibus (28) or the EBI ArrayExpress (29). Search terms included ‘suprachiasmatic’, ‘SCN’, ‘adult’, ‘wild-type’ and ‘whole brain’. Only using data where MIAME (Minimum Information About a Microarray Experiment, https://www.ncbi.nlm.nih.gov/geo/info/MIAME.html), has been supplied and where FLEO is possible (i.e. those with .CEL files available). Whole brain data gathered usually consisted of the control (or sham) samples without/before treatment, but not those within treatment groups. Likewise, SCN data were restricted to sham treatments and wild-type animals. The majority of the whole brain data reported the inclusion of the cerebellum and olfactory bulbs in samples. We limited the comparison to adult mice, but with no restriction on strain or sex. The microarray data used in the current study are detailed in Table 1 (a more detailed in version of this table can be found in Supplementary File 1).

**Table 1.** Data collected for the meta-analysis as a series of comparisons (studies) within Affymetrix microarray platforms and RNA-Seq data

Accession code	File codes	SCN or WB	PMID	Arrays/ Samples	Study: Platform
GSE6904	GSM159292–4	SCN	18021443	3 SCN vs 8WB	1: 430AV2 Mouse
GSE7814	GSM189596/598/600/602	WB	17991715		
GSE7814	GSM189628/630/632/634	WB	17991715		
GSE16496	GSM414571–2	SCN	21858037	3 SCN vs 13WB	2: 430V2 Mouse
GSE28574	GSM707557	SCN	21610730		
GSE20411	GSM511616–20	WB	20526689		
GSE20411	GSM511621–25	WB	20526689		
GSE9954	GSM252077–9	WB	18365009		
MEXP-3933	WT_sham_1–6	SCN	23993098	6 SCN vs 22WB	3: MoEx 1.0ST
GSE27282	GSM674605–14	WB	21625610		
GSE27282	GSM674615–26	WB	21625610		
E-MEXP-3493	WT_ZT6(1to4), WT_ZT11(1to4), WT_ZT13(1to4)	SCN	22264613	12 SCN vs 12WB	4: MoGene 1.0ST
GSE34469	GSM849761–2, GSM933084–5	WB	23580197		
GSE34305	GSM847026–7/29/31/33	WB	22560501		
GSE24940	GSM613009–11	WB	21088282		
PRJEB9284	SAMEA3368226-SAMEA3368230, SAMEA3368237	SCN	26232227	7 SCN vs 10WB	5: RNA-Seq
PRJNA235222	SAMN02585109	SCN	24531307		
GSE43013	GSM1055111	WB	25677554		
GSE30352	GSM752614	WB	22012392		
GSE30352	GSM752615	WB	22012392		
PRJEB2494	SAMEA811980	WB	PMC3428933		
PRJEB2494	SAMEA811978	WB	PMC3428933		
GSE41338	GSM1015150/51	WB	23258891		
GSE41637	GSM1020640	WB	23258890		
GSE41637	GSM1020649	WB	23258890		
GSE41637	GSM1020657	WB	23258890		

The original experiments from which the data can be tracked by database codes or Pubmed IDs (references 91–103). A more detailed and extended version of this table can be found in the **Supplementary File 1**.

### Feature level extraction output (FLEO)

Microarray data were processed as series of comparisons for each microarray platform, or RNA-Seq data. CEL files were imported in to AltAnalyze (version 2.9.0.2) and processed via integration with Affymetrix power-tools (APT), using the Robust Multi-array Averaging (RMA-sketch) algorithm. Following removal of probes known to cross-hybridize, the remaining probe-sets were then mapped to build 72 of the ENSMBL mouse database (GRCm38.p1). For exon-arrays (Affymetrix MoEx 1.0ST) expression at the gene level was examined, with this achieved using only those probe-sets that match to exons present in all expressed known splice variants of the ENSMBL transcript in question (constitutive probe-sets). For the comparisons of 3′ arrays (430v2 and 430Av2), those probe-sets known to cross-hybridize (_x_ and _s_ -appended) and those without mappings being excluded. The average values of Hedges’ g and Variance were calculated for each gene symbol or ENSMBL GID.

### Microarray pre-processing for meta-analysis

Effect sizes (ES) for each comparison were calculated as Hedges’ g values (35). Briefly, this involves calculation of Cohen's d value (log2 fold-enrichment SCN vs WB, divided by pooled standard deviation), followed by an adjustment of number of arrays (known as the j factor). The variance of each ES was also calculated.

### RNA-Seq pre-processing for meta-analysis

Published RNA-Seq projects were obtained from the Sequence Read Archive (Table 1, and in more detail in Supplementary File 1). Sequence reads were aligned to the mm10 genome using TopHat2 (36). When unpublished, library strand direction was confirmed with manual inspection in the Integrative Genomics Viewer (37). Junction BED files were sent to AltAnalyze for downstream gene annotation, counting and differential expression analysis (38). Per-identifier ES values were then calculated as described above.

### Meta-analysis

All data were indexed against MGI gene symbols (using BioMart mouse version 72), with a total of 31 684 MGI gene symbols having data for at least one platform. The combined effect size was calculated as described for the inverse variance methods described by Choi *et al.* (33). We calculated effect sizes and significance for the inverse variance meta-analysis based on a random effects model (REM), as this does not assume that there is a single common effect size, but rather a range of true effect sizes with additional sources or variation. Basic calculation and indexing was carried out from the output of AltAnalyze for each study. The analysis was carried out using the PyData stack in the Python programming language (version 2.7.11, as part of the Anaconda python distribution version 4.0.0, from https://www.continuum.io/downloads and is available as a series of interactive notebooks (at https://github.com/LozRiviera/SCN_enrich_Meta, DOI: 10.5281/zenodo.324907). From the meta-analysis, *Z*-values were used to calculate *P*-values from two-tailed tests and subsequently to apply a multiple-testing correction in the form of the false discovery rate (FDR) *q*-value (39). The subsequent FDR-adjusted *q*-values were calculated from *P*-values in R (version 3.2.2, (40)), using the ‘qvalue’ package (version 2.0) (41).

### Resources to examine the distribution of SCN-enriched transcripts

Confirmation of potential SCN-enriched genes using online resources involved searches by gene symbol on both the Allen Brain Atlas (42) and the GENSAT database (43) .

### Immunohistochemistry

All work was carried out in accordance with Animal [Scientific Procedures] Act 1986, with procedures reviewed by the clinical medicine animal care and ethical review body (AWERB) for the University of Oxford, and conducted under project licence PPL 30/2812 and personal licence IDB24291F. Young-adult (8–24 weeks of age) male wild-type C57BL/6J mice (RRID:IMSR_JAX:000664), were obtained from Envigo (Alconbury UK) and housed in specific pathogen free conditions, with the only reported positives on health screening over the entire time course of these studies being for *Helicobacter hepaticus* and *Entamoeba spp*. All animals were singly-housed, provided with food and water *ad-libitum* and maintained on a 12-h light:12-h dark cycle (150–200 lux, cool white LED, measured at the cage floor), in light-tight environmental enclosures.

Brains were fixed by perfusion with (then immersion overnight in) 4% paraformaldehyde in phosphate-buffered saline (PBS). Following cryoprotection in 30% sucrose in PBS, brains were embedded in optimal cutting temperature compound (OCT, VWR International Ltd.) and sectioned at a thickness of 14–20 μm. The primary antibody for SYTL4 (1:200–1:500, Rabbit polyclonal, ab110519, Abcam plc, Cambridge, UK, RRID:AB_10858160) was used following blocking with 10% Donkey serum in PBS + 0.1% Triton X-100 + 0.1% Tween 20 (serum and detergents from Sigma-Aldritch Ltd., Dorset, UK). Validation of the primary antibody is included in Supplementary File 1. Detergents were excluded following the blocking step to prevent loss of lipid micro-domains due to excessive permeablization. The secondary antibody was Donkey anti-rabbit Alexa Fluor^®^ 568 conjugate (1:200, Thermo-Fisher Scientific Inc.). Confocal images were collected using a Zeiss LSM710 (Carl Zeiss Ltd., UK).

### Gene ontology (GO) and network analysis

Data were imported into Cytoscape (44) (version 3.3.0). Established protein–protein interactions were obtained from the STRING database (45) (http://string-db.org), using the list of both enriched and depleted genes (gene symbols) and the StringApp plugin for Cytoscape (version 0.9.2). The BiNGO plugin (version 3.0.3) (46) was used to show gene ontology (GO) terms that are over-represented when compared by Hypergeometric test to the whole GO annotation, following Benjamini–Hochberg false discovery rate (*α* = 0.05). GO (47) files and MGI annotations were obtained as of 4 February 2016 and the tests were run using gene symbols. Visualization of the results of the ontology analyses were carried out using the Enrichment Map plugin for Cytoscape (version 2.1.0) (48), with defaults for BiNGO files (p-cutoff = 0.001, q-cutoff = 0.05, Jaccard similarity cutoff = 0.25).

## RESULTS

### Identification of SCN-enriched and SCN-depleted transcripts

Restricting data to MIAME-compliant datasets where Affymetrix CEL. files were deposited, a total of 79 microarrays were obtained (24 SCN and 55 WB), from four different microarray platforms, alongside 17 RNA-Seq data files (7 SCN and 10 WB). These data are summarized in Table 1 (and in more detail in Supplementary File 1). Indexing to MGI gene symbols resulted in 31 684 symbols having data for at least one platform, with the number of symbols covered by each platform and the degree of overlap between platforms shown in Supplementary File 1 and Table 1.

**Figure 2.**
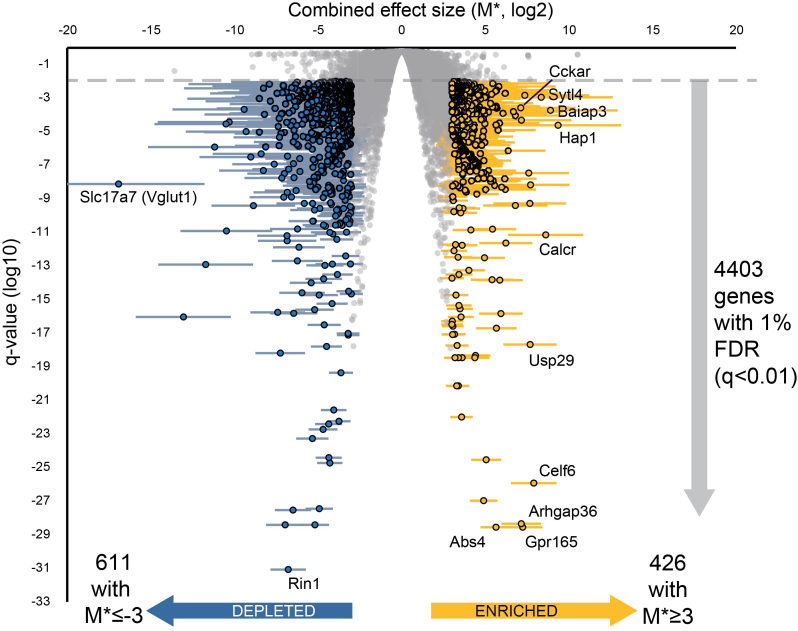
Volcano plot of genes found to be enriched in the SCN by meta-analysis. Plot shows enrichment in SCN versus WB (combined effect size, M*, derived from log_2_-transformed fold changes) against the significance (FDR-corrected *q*-value), with error bars showing 95% confidence intervals. Genes with *q* < 0.01 and M* ≥3 shown in gold (426 highly enriched) and those with *q* < 0.01 and M* ≤−3 shown in blue (611 highly depleted). Genes labelled are the top enriched genes by combined effect size, or by significance, as well as the most depleted and most significant depleted genes.

Available data were processed as five studies based on the microarray platform (or RNA-Seq). The meta-analysis was conducted using an REM for weighting individual effects scores. These scores were themselves derived from the differences in expression between the SCN and WB samples in each study (log_2_-transformed fold-changes). The data are summarized in Figure 2 and is also implemented in an interactive version to improve the accessibility of the data (Supplementary File 2). All MGI gene symbols with at least one datum (study) can be found in the Supplementary File 3. Constraining the results using a positive false discovery rate (pFDR) *q*-value of 0.01 (expected that around 1% of results are false-positives) gives a list of 4403 symbols. Where discussed in the remainder of the paper, only genes passing this pFDR correction will be referred to as ‘enriched’ or ‘depleted’. To obtain a manageable list of genes for pathway analysis these 4403 genes were further restricted to those with and a combined effect size (M*) of 3 or more in either direction (Figure 2), leaving a list of 1037 gene symbols. Of these, 426 were enriched and 611 depleted.

The top 20 enriched gene symbols from meta-analysis (ranked by combined effect size) are presented in Table 2, with corresponding evidence from online resources for gene expression. http://www.alleninstitute.org (42) and http://www.gensat.org/ (43). It is notable that many of the genes associated with the known function of the SCN, such as canonical clock genes, are not present in this list. This is in part due to the nature of the study, with both expression in other nuclei in the brain and variations in expression over time (rhythmic or otherwise) being likely to increase variation for such a transcript, therefore decreasing its enrichment score. It is the case that many genes such as *Vip* show combined effect sizes well above zero (see Table 3, a table of genes with established roles in the SCN), but are excluded from the current list of 4403 genes by *q*-value.

**Table 2.** Top 20 results from meta-analysis (ranked by combined effect size), with corresponding evidence from online resources for gene expression

MGI gene symbol	Description	Number of studies	Combined effect size (M*)	pFDR *q*-value	Allen Brain Atlas	GENSAT
Hap1	huntingtin-associated protein 1	5	9.36	2.42E-05	14890	91396
Baiap3	BAI1-associated protein 3	5	8.90	1.87E-04	75081206	85221
Calcr	calcitonin receptor	5	8.61	7.17E-12	12096	60456
Sytl4	synaptotagmin-like 4	5	8.35	1.13E-03	75651223	x
Celf6	CUGBP, Elav-like family member 6	4	7.91	1.18E-26	71358616	66740
Ahi1	Abelson helper integration site 1	5	7.71	6.84E-09	69549642	x
Usp29	ubiquitin specific peptidase 29	2	7.68	2.14E-18	70194636	x
Ngb	neuroglobin	5	7.67	5.38E-10	79556712	x
Zcchc12	zinc finger, CCHC domain containing 12	4	7.60	3.42E-08	73817424	80305
Vwa5b1	von Willebrand factor A domain containing 5B1	3	7.36	1.47E-03	51559	x
Arhgap36	Rho GTPase activating protein 36	4	7.24	2.80E-29	69352834	x
Gpld1	glycosylphosphatidylinositol specific phospholipase D1	5	7.16	4.88E-05	74509585	x
Gpr165	G protein-coupled receptor 165	4	7.15	4.54E-29	70560278	75666
Cckar	cholecystokinin A receptor	5	7.12	2.62E-04	203	x
Tmem130	transmembrane protein 130	4	6.80	4.03E-10	89067	x
Chodl	chondrolectin	5	6.80	9.14E-05	71380977	x
Itih3	inter-alpha trypsin inhibitor, heavy chain 3	5	6.69	1.58E-04	600	x
Nap1l5	nucleosome assembly protein 1-like 5	5	6.38	7.34E-07	72080123	x
Scn9a	sodium channel, voltage-gated, type IX, alpha	5	6.23	2.34E-12	71325438	x
RP24–361E14.1	x	1	6.20	3.62E-09	x	x

http://www.alleninstitute.org and http://www.gensat.org/. ‘x’ indicates no data or identifier currently available.

**Table 3.** The enrichment of previously suggested SCN markers and genes important for signalling or rhythmicity, along with the combined effect size (M*) derived from the meta-analysis

MGI gene symbol	Effect size (M*)	Pubmed ID
*Adcyap1* (PACAP)	2.20	9065523
*Avp*	**4.93**	25741730
*Drd1a*	−2.32	25643294
*Lhx1*	2.52	21525287
*Nms*	**3.86**	15635449
*Prok2*	**3.19**	12024206
*Rgs16*	**3.11**	21610730
*Scg2*	**4.70**	17319750
*Six3*	**3.85**	21525287
*Six6*	**5.67**	21525287
*Syt10*	**3.29**	21921292
*Vip*	6.12	11207820
*Vipr2*	**2.48**	12086606

Values in bold are those that meet the relevant cut-off (*q*-value < 0.01). Pubmed IDs provided for examples of the many references suggesting functional involvement in the SCN.

### Validation of an SCN-enriched transcript, Sytl4

**Figure 3.**
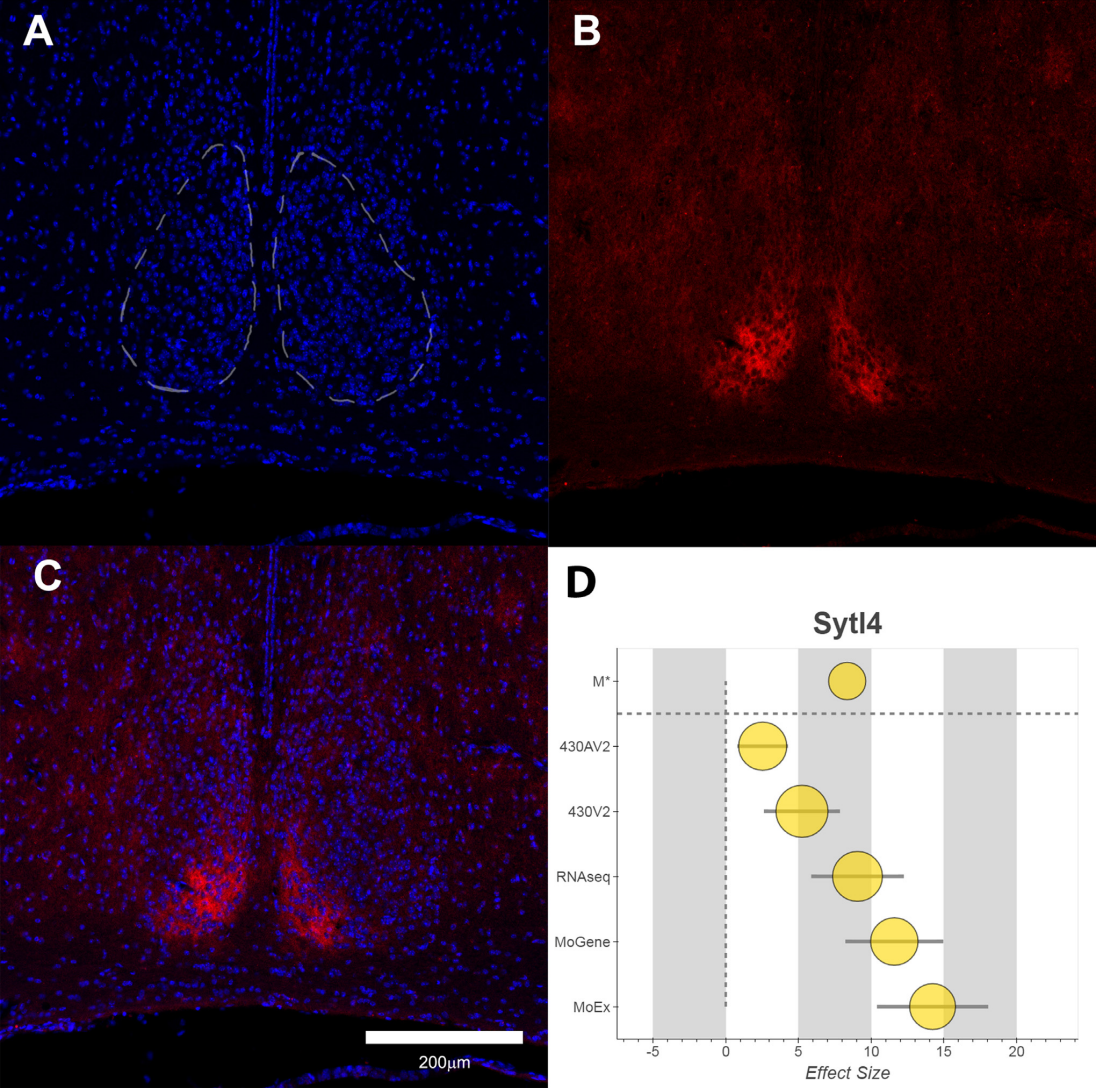
Distribution of the SYTL4 protein in the Hypothalamus. (**A**) DAPI staining of Nuclei shown nuclear-dense SCN and surrounding tissue. (**B**) SYTL4 shows strong expression in the dorsomedial SCN. (**C**) Merge of both channels. (**D**) Forest plot of the data in the meta-analysis for *Sytl4*, consisting of the combined effect size (M*, top), followed by individual Hedges’ *g* values for each platform/study. Error bars show variation within each study and node size represents the weighting towards the final M* value. The tissue in this figure was from a C57BL/6J male mouse, collected at ZT18.

It is possible that the combined effect size alone may be a good indicator of genes with important roles in the function of the SCN. To examine this further, we selected another gene with a high M* value (8.35, ranked fourth by M* in list of 4403 gene symbols at 1% pFDR) that has not previously been described in the SCN, *Synaptotagmin-like 4* (*Sytl4*, or *Granuphilin-a*). The protein is known to be involved in the controlling of the release of dense-core vesicles and exosomes, but has no known role in the SCN to date. Immunohistochemistry on fixed SCN tissue revealed that the SYTL4 was indeed expressed in the SCN, as well surrounding hypothalamic nuclei and was largely absent from the rest of the brain (see Figure 3, an example of the distribution of SYTL4, from tissue collected at ZT18). The main known binding partner of SYTL4, RAB27A, is also enriched in the SCN (Rab27a, M* = 3.40) (49).

### Pathway analysis

**Figure 4.**
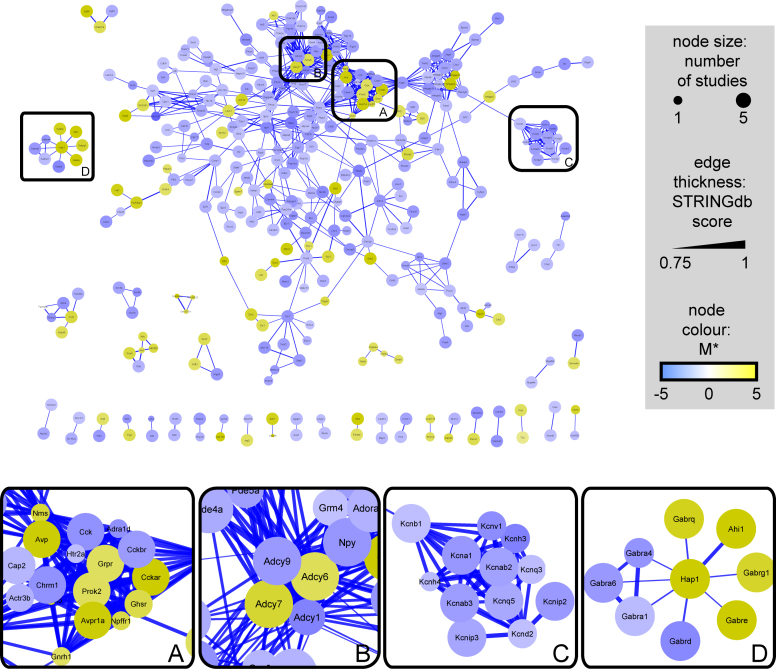
Network graph of SCN-enriched and SCN-depleted transcripts, constructed using interactions from the STRING database. Cut-off for interactions was an overall STRING score ≥0.75 and only those gene symbols with one or more interactions are displayed (339 of 1037). Side Panels (**A**–**D**) are enlargements of clusters within the network. Nodes are shown in gold for enriched transcripts and blue for depleted ones, with edge-thickness mapped to the overall score of the interaction from the STRING database (thickest lines = highest confidence).

**Figure 5.**
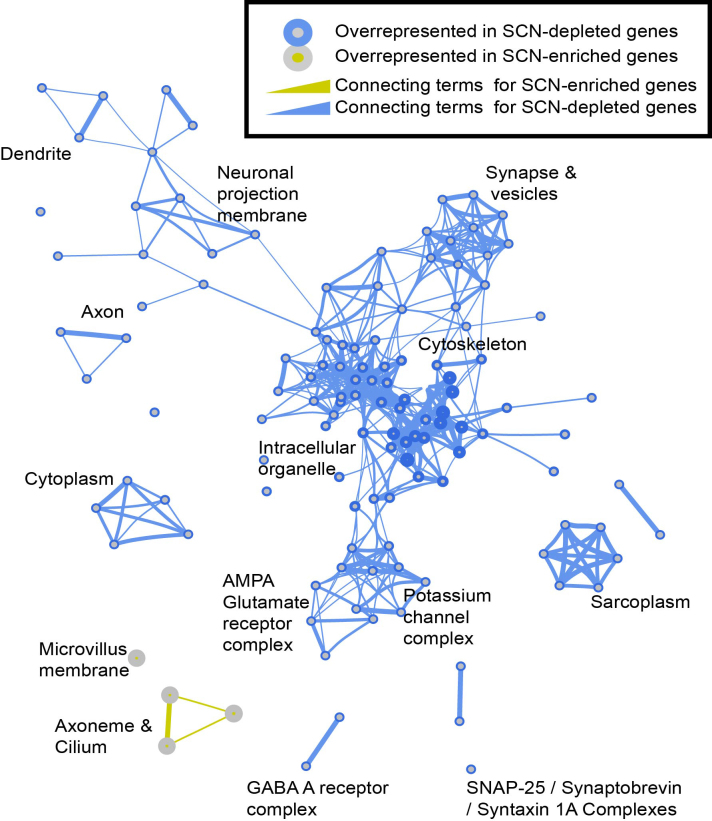
Enrichment map of the gene ontology data for SCN-enriched and SCN-depleted transcripts. Enrichment map generated from BiNGO output file for cell components (output from BiNGO analysis provided in Supplementary File 4).

To further examine the potential relationships between the 1037 transcripts, both enrichment of GO terms and known interactions were examined. Figure 4 shows known interactions (of high confidence) between those gene symbols in the list of 1037, as revealed by the STRING database. Like much of the brain, the SCN relies on a balance of both glutamatergic and GABAergic neurotransmission and as such the levels of some transporters for glutamate and GABA differ greatly. The vesicular GABA transporter VGAT (encoded by *Slc32a1*) is consistently enriched throughout all the studies in the meta-analysis (M* = 3.41), whereas the vesicular glutamate transporter VGLUT1 (*Slc17a7*) is the most depleted transcript in the SCN when compared to the brain on the whole (M* = −16.93). Peptidergic transmission is also known to play a vital role in the SCN and other hypothalamic nuclei, (Figure 4, **box A**.), with the enrichment of GO terms suggesting that the axoneme and primary cilium, a focal point for GPCR-mediated signalling (50), may also be important for communication between cells in the SCN (Figure 5). Where families of genes/proteins are known to be important in the tissue of interest, a list of enriched transcripts may help identify the most relevant isoforms for further research. In the case of adenylate cyclases it seems that the SCN shows enriched expression of isoforms 6 and 7 and substantial depletion of isoforms 1 and 9 in particular (Figure 4, **box B**.). One clear finding from pathway and ontological analysis is that substantial parts of the synapse and many voltage-gated channels are depleted in the SCN. This is shown in the interactions drawn from the STRING database for K^+^ channels (Figure 4, **box C**) and throughout the enrichment of GO terms related to both axons and dendrites (Figure 5). Additionally, whilst the neurotransmitter GABA and its receptors are ubiquitously expressed throughout the CNS, in the SCN the expression of subunits that comprise GABA-A receptors suggest that the stoichiometry may differ from much of the rest of the brain (Figure 4, **box D**). The complete lists of enriched GO terms is provided as Supplementary File 4.

## DISCUSSION

The SCN provides a fascinating interplay between intracellular molecular clocks and the synchronized daily rhythms of neuronal activity (4,51). Although this rhythmic control over neuronal activity continues in the absence of external influences, the circadian pacemaker must be capable of responding to photic cues as well as inputs from other brain regions. These processes may involve a different set of genes and proteins, not all of which are expected to be rhythmically expressed. The current study helps to identify those transcripts, rhythmic or otherwise, that are found in the SCN more often that in the brain as a whole. We have shown that 4403 genes are expressed at a significantly higher (enriched, 2346 with *q* < 0.01) or lower level (depleted, 2057), when compared to the brain as a whole. Furthermore, the approach taken uses only existing public data and would continue to grow in strength as more data are deposited in public repositories.

This meta-analysis has provided a robust list of transcripts that are differentially expressed in the SCN when compared to the rest of the brain. This includes transcripts which are selectively enriched in the SCN which provide new avenues for understanding SCN physiology. Amongst the transcripts found to be selectively enriched in the SCN are genes already known to play a role in SCN function or circadian timing, or transcripts that have been used as markers of the SCN (Table 3). These transcripts include the genes for well-established peptide neurotransmitters such as arginine-vasopressin (*Avp*), Neuromedin S (*Nms*) and Prokineticin 2 (*Prok2*) (52–54). In addition to signalling molecules, genes such as *Ngb*, and *Zfhx3* are highly expressed in the SCN. Knockout of Neuroglobin (*Ngb*, M* = 7.67), increases responses of the SCN to light-pulses via the retina (55) and *Zfhx3* is a zinc-finger homeobox domain gene, recently shown to act via AT motifs on many neuropeptide promoters (9).

The focus of the current study was to look at over-represented transcripts in the SCN, rather than rhythmic ones. As summarized in Figure 1B, SCN samples were taken from a range of times (both ZT and CT), with some of the samples pooled prior to measuring transcription, therefore making it difficult to further assess rhythmicity. Indeed, the methodology may select against those genes that cycle in expression as this will increase the variation in these data. An example is the vasoactive intestinal polypeptide gene (*Vip*), a neuropeptide transmitter with an established role in the SCN(19). Despite a high combined effect size (M* = 6.12), *Vip* is excluded from the list of enriched transcripts due to high variability (FDR *q*-value > 0.01), as is Magel2 (M* = 7.67). Mice lacking *Magel2* show normal rhythms and entrainment, but with a reduced amplitude and increased daytime activity and MAGEL2 is expressed predominantly within AVP-containing neurons in the SCN (56,57). There may be further genes with relevance to the SCN that show high M* values, but that are excluded due to their variability. Furthermore, with this selection against variable transcripts in mind, along with the fact that the core ‘transcription-translation feedback loop’ (TTFL) is expressed in cells throughout the body, it is not surprising that elements of the TTFL are not enriched in the current study (none of the 4403 transcripts with a *q* < 0.01). Some genes with roles in circadian rhythms are depleted (*Cry2*: M* = −3.30, *Npas2*: M* = −2.95, *Bhlhe40 (Dec2)*: M* = −2.89) when compared to expression in the whole brain.

Although the primary interest of this study is enriched transcripts, the analysis also identified transcripts that appear to be selectively depleted in the SCN. One notable finding is that large numbers of immediate early genes (IEGs) are found to be highly depleted in the SCN (Supplementary File 1, Supplementary Table 2). IEGs are important for converting experience to changes in plasticity in the brain, including the transcriptional responses to events such as light-pulses (58–60). Our findings might suggest that expression of IEGs is not required for circadian rhythmicity in the SCN and that the activation of IEGs from a low baseline of expression may be vital for external stimuli to modify phase and plasticity in the SCN.

### Intra-neuronal communication and synchrony in the SCN

The SCN is comprised of a network of cellular oscillators and the balance between synchronising and phase-repulsive signalling is essential for coordinated output from the circadian pacemaker (61,62). The density of synaptic connections (synapses per cell) has been reported as sparse (21,63) and the densely-packed neurons of the SCN possess smaller cytoplasmic compartments and reduced axonal and dendritic arborizations when compared to other neurons throughout the brain (64). These reports are in accordance with the overrepresentation of ontological terms such as ‘synapse’ and ‘dendrite’ within depleted transcripts (Figure 5 and Supplementary File 4) supporting this lack of extensive neuritic morphology in the SCN. The list of enriched genes contains many signalling molecules and their receptors that are already known play roles in coupling the cells of the SCN together (see Table 3). Proteomic analysis has previously pointed to the cycling of synaptic vesicles as essential for robust circadian synchrony (65). The importance of dense-core vesicles in a functional SCN is also indicated by the finding that double-knockouts for the transmembrane proteins of these vesicles, *Ptprn* (ia-2) and *Ptprn2* (ia-2β), show a loss of diurnal rhythms in heart-rate, temperature and locomotor activity (66). Many of the SCN-enriched transcripts identified in this study have no known role in the circadian pacemaker. One such gene, *Sytl4*, has been shown to play an important role in regulated exocytosis, controlling the release of insulin and other substances from dense-core vesicles in tissues and cell lines (67,68). Other studies have reported hypothalamic expression and a role in facilitating sexually dimorphic behaviours in the hypothalamus (69). Immunohistochemistry in the current study confirms the enrichment of SYTL4 in the SCN, supporting the role of dense-core vesicles in SCN function.

In addition to the peptidergic communication, GABA-A receptor mediated signalling is known to be important to the functioning of the SCN, in particular the ability of GABA to promote both synchrony and disorder (21,62,70). Unlike many other areas of the brain, in the SCN it seems that some of the less-studied GABA subunits (*Gabre* and *Gabrq*) may play important roles, alongside the expression of multiple GABA transporters. This analysis also suggests that subunits of the recognized ‘extra-synaptic’ receptors (e.g.*Gabrd*, M* = −8.87) are selectively depleted in the SCN. Genes relevant to the function of GABAergic signalling in the SCN also extend to known interactors such as *Hap1*, which has been shown to control recycling of GABA-A receptors to the synapse, preventing internalization and recycling (71). *Hap1* (Huntingtin-associated protein 1) was discovered as a binding partner for Huntingtin and is therefore implicated in the neurodegenerative disorder Huntington's disease (72). These findings are of particular interest considering the circadian abnormalities reported in both human and animal models of this disease (73) and may be an important part of understanding GABAergic signalling, both from the SCN and within the SCN, where release of GABA can be either brief, or tonic over many hours (61).

### Imprinting and SCN function

A number of the transcripts that show enrichment in the SCN are known to be imprinted (allelic bias towards either the maternal or paternal copy of the gene in question). For example imprinted genes such as the E3-ubiquitin ligase, *Ube3a* and the G-protein *Gnas* have effects on the stability of circadian rhythms and sleep architecture (74–76). Although these genes are not present in the list of 4403 enriched and depleted genes, when a list of 45 confirmed imprinted genes (77) was matched to these 4403 genes in our study (43 symbols matched) 13 of these genes were present (see Supplementary File 1, Supplementary Table 3). This is significantly more than would be expected by chance (*P* = 0.0044, 13 or more from 43 being picked in 4403 from a total pool of 31 684, hypergeometric distribution). The presence of 12 imprinted genes in the list of those substantially and significantly enriched in the SCN (of the 426 genes with M* >3 and *q* < 0.01) is even more significant (*P* = 2.2e-11, 12 or more from 43 being picked in 426 from a total pool of 31 684, hypergeometric distribution). Although the true extent of imprinting is still the subject of some discussion, recent papers have reported that parental allelic bias during development is often accompanied with a higher overall expression (78,79). These findings raise the interesting possibility that parent of origin effects may occur in the molecular mechanisms underlying mammalian circadian rhythms.

### New pathways and implications

Recently, a number of consortia have released the first genome-wide association studies concerning circadian and sleep profiles (80,81). Genes common to both these studies include known circadian genes, including *Per2 and Rgs16* (M* = 3.11), as well as others, such as *Ak5* and *Erc2*, which are significantly depleted in the SCN in the current study. In a similar way to that of the IEGs, this may indicate the potential for activation from a low resting level of transcription and does not preclude these genes playing important roles in the SCN.

### Investigating uncharacterized transcripts

Another consideration for an ongoing meta-analysis to identify tissue-enriched genes is that it provides a strong statistical backing for investigating those transcripts that have received little or no previous attention. Researchers tend to focus on genes and proteins where some research has been recorded previously, and understudied transcripts and genes represent large gaps in knowledge (82,83). Certainly, in the case of these functionally enigmatic genes in the brain, the gaps in knowledge are an effect of when the genes were discovered, rather than any additional complexity or novelty in the genomic or protein sequences (84). As further data are added to such meta-analyses, increasing certainty about expression should lead to study of such transcripts. Now that deposition of transcriptomic data has become part of the established scientific publication process it is likely that meta-analyses like the one described here will continue to grow in power and utility. Furthermore, it seems that this may also be a valuable way to make use of the many well-executed but underpowered microarray and RNA-Seq experiments that may exist in laboratories without ever having reached publication.

### Technical considerations

It is important to clarify that the results are heavily-dependent on the way the question has been approached, in that we have compared SCN to the rest of the brain and have prioritized stable transcripts across all studies. Transcripts that show high levels of expression in the SCN and also in other regions of the brain may not be detected as enriched. The same is true of transcripts that cycle rhythmically, even though they may have a peak of expression that would see substantial enrichment in the SCN. With any microarray experiment, it is important to consider the potential sources of error. Aside from the risk of false positives within the significant results the most likely reason for incorrect reporting of SCN-enrichment is likely to be contamination of the sample by surrounding hypothalamic nuclei. The top 20 list of enriched genes contains a number of genes where the hypothalamic pattern of expression is not restricted solely to the SCN (as shown in the data from GENSAT and the Allen Brain Atlas). This may be a reflection of the number of experiments that made use of tissue-punches to isolate the SCN. This technique prioritizes faster isolation of RNA with lower risk of degradation, whereas the use of laser-capture microscopy favours accuracy, but risks bias introduced by uneven sampling across the whole SCN. SCN tissue collected by both methods has been utilized in the meta-analysis.

The variation in the data we have used in the current meta-analysis consists of variation within each of the studies we have defined (SCN and WB data from the same transcriptomic platform) and variance between the studies (τ^2^). Whereas the variance in individual studies decreases with the number of samples (array files or RNA-Seq samples), τ^2^ will only be decreased with an increasing number of studies. In this respect, limiting the design to one large study per array/transcriptomics platform increases the effects of the between-studies variation. It may be that the costs of such an approach are particularly high when looking at the SCN, where the size and temporal variation in transcription will likely increase the biologically relevant variation between studies. Recent discussions have suggested that, where τ^2^ is large, a Fixed Effects Model could be used for the meta-analysis, or more complex Bayesian models could be explored (85). However, with many rhythmic genes present in the data, the large variation between studies may be a sign these genes are both rhythmic and enriched overall. Furthermore, the intent of the current work was to look at enrichment without the assumptions inherent with complex tools or statistical models. I^2^ values in the data table (see Supplementary Files 2 and 3) indicate the amount of variation that is not explained by variation within studies (heterogeneity or inconsistency in the meta-analysis (86)) and these may be a better guide to the biological variation of a given transcript (including circadian rhythmicity).

As previously discussed, factors such as the sex, age and strain of the mice used might be expected to change to outcome of the study. Certainly changes in SCN gene expression have also been shown to occur with age (87). For example, SIRT1 levels decline with age, which has been shown to be related to the reduced stability of the molecular clock (88). Sex differences may also alter the function of the SCN (89) and underlying transcription, as could the background strain of the mice used. However, the majority of the samples used for both SCN and WB are from young male mice (up to 16 weeks of age) and no consistent disparity between SCN and WB samples exists in all the studies. Furthermore, accepting these sources of variation into an inclusive meta-analysis is likely to highlight those genes that are consistently enriched in the SCN, regardless of other variables. As more SCN transcriptomic data become available, it may be possible to conduct meta-analyses which further partition data to allow both rhythmic and SCN-enriched genes to be identified. Genes which show strain, age or sex-related differences in the SCN could be investigated in a similar manner. However, the currently available data is insufficient to allow these factors to be reliably addressed. As such, a comparison of existing data resources to identify rhythmic genes (e.g. CircaDB, Pizarro *et al.* (90)) combined with the SCN enrichment data described here is perhaps the best approach to identify candidate genes by both their circadian expression and SCN enrichment.’

## CONCLUSION

Meta-analysis of transcriptomic data provides a powerful approach to identify enriched and depleted transcripts in specific brain regions. Here we apply this approach to the SCN, the master circadian pacemaker of the mammalian hypothalamus. Although many tissues display rhythmic transcription, those transcripts that are consistently enriched in the SCN are likely to have important functions in the generation and maintenance of circadian rhythms. The current analysis has identified both transcripts previously reported as highly expressed in this brain region and genes known to play key roles in SCN physiology, although no single transcript is revealed with high levels of expression restricted solely to the SCN. Moreover, this study identifies a range of transcripts that have, to date, received little attention and have no known SCN function. These provide *bona fide* candidates for future studies to further understand the molecular basis of co-ordinated biological timing.

## Supplementary Material

Supplementary Data
