# Supplementary material for: Meta-analysis of transcriptomic datasets identifies genes enriched in the mammalian circadian pacemaker
Source: Nucleic Acids Res. 2017 Aug 18;45(17):9860–73. doi: 10.1093/nar/gkx714 (PMC5737434; doi:10.1093/nar/gkx714)
Supplement: Supplementary Data [file gkx714_supp.zip › nar-00706-n-2017-File007.pdf]

Supplementary methods: Equations and addtional resources

A: Calculations of the effect size for each study (microarray/RNA-Seq platform)

Using Hedges' g value, an adjusted Cohen's d value

$$Enrichment = \bar{X}_1 - \bar{X}_2$$

where group1 is the values for expression in the SCN and group2 those for the Whole Brain

(SCN mean - WB mean) (Logged values, so subtracting values gives ratio)

$$Pooled\ Standard\ Deviation = \sqrt{\frac{(n_1 - 1)S_1^2 + (n_2 - 1)S_2^2}{(n_2 + n_2) - 2}}$$

Where: S = standard deviation of the group and n = number of data in the group

$$Cohen's\ d\ value = \frac{Enrichment}{Pooled\ Standard\ Deviation}$$

$$Correction\ Factor(J\ Factor) = 1 - \frac{3}{4df - 1}$$

where **df** = degrees of freedom (total data, SCN plus WB, -1)

$$Hedges'\ g\ value = Cohen's\ d \text{ x } J$$

$$Variance\ in\ d(V_d) = \frac{n_1 - +n_2}{n_1 n_2} + \frac{d^2}{2(n_1 + n_2)}$$

$$Variance\ in\ g(V_g) = J^2 \text{ x } V_d$$

$$Standard\ Error\ in\ g(SE_g) = \sqrt{V_g}$$

B: Equations used to calculate combined effect size (M*), using a Random Effects Model (REM)

es = effect sizes, imported from indexed file (Hedges g)

W = weight (inverse of Variance in Hedges g)

$$\frac{1}{var}$$

M* = Combined (mean) effect size

W* = weighting in random effects model (inverse of study variance + between studies variance)

$$\frac{1}{var + Tau^2}$$

With

$$Tau^2 = \frac{Q - df}{C}$$

Where:

df = degrees of freedom (number of studies -1)

$$Q = \sum es^2 * W - \frac{(\sum es * W)^2}{\sum W}$$

$$C = \sum W - \frac{\sum W^2}{\sum W}$$

and I squared statistic (heterogeneity within the meta-analysis, see Higgins et al 2003 BMJ, 327:557-60)

$$I^2 = \frac{Q^2 - df}{Q}$$

The Interactive notebooks linked to in the manuscript constitute the meta-analysis carried out in the paper

To repeat the analysis:

- unpack the copies of the Altanalyze output
- run all calculations of 'Hedges g' (A1-5)
- index and combine the values in files from A1-5 (B1)
- then finally run the REM meta-analysis (B2), followed by the vizualisation (C) if required

Supplementary methods II: Characterization of the antibody for mouse SYTL4 (abcam ab110519,RRID:AB_10858160)

NIH-3T3 cells were plated in a 96-well plate at $4x10^4$ cells/well (60-70% confluence), with 5pmol of siGENOME SMARTpool siRNAs (Dharmacon, GElifescences, USA) targeting either mouse *sytl4* (Code: M-040841-00-0005) or pooled non-targeting control siRNAs (code: D-001206-13-05) applied to the plated cells after 24hrs. 42hrs after addition of the siRNAs, all cells were fixed (4% Paraformaldehyde in phosphate buffered saline, PBS) for 1 hr at 20^0C. Blocking and permeablization with 10% Donkey serum in PBS + 0.1% Saponin (Sigma-Aldritch, UK), was followed by staining with primary antibody for SYTL4 (1:200) rabbit polyclonal, ab110519, Abcam plc, Cambridge, UK, RRID:AB_10858160). The secondary antibody was Donkey anti-rabbit Alexa Fluor® 568 conjugate (1:200, Thermo-Fisher Scientific Inc.), applied for 1hr at 20^0C and followed by three PBS washes. Images were collected from the centre of each of three wells for each siRNA with identical settings on IX81 inverted microscope, using CellSense Software (Olympus KeyMed Ltd, UK). All wells were confluent (close to 100% coverage of field) at the time of fixation. All images were processed in parallel and the mean intensity of each image was used to compare conditions.

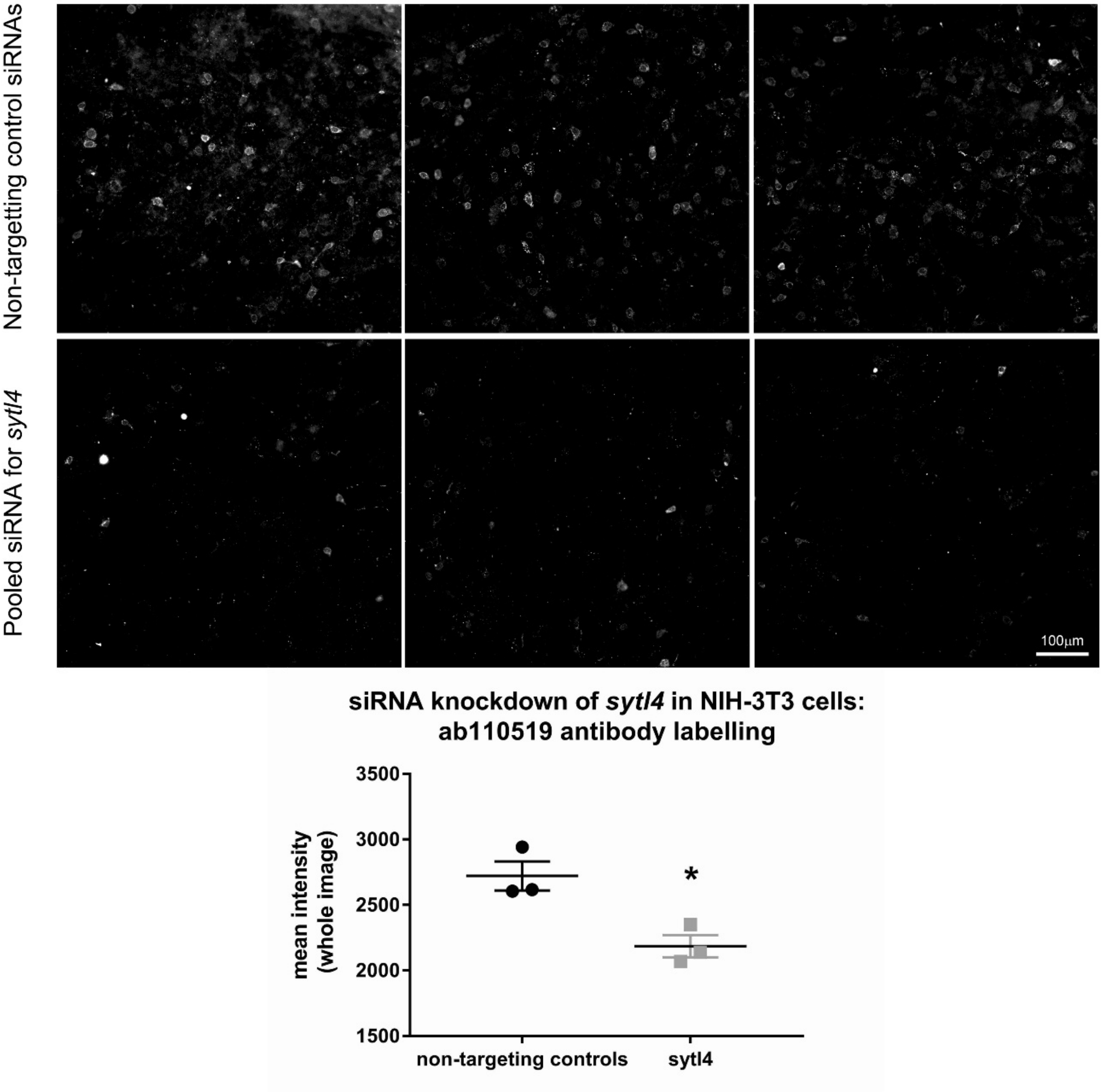

Figure: Top panel – Images from 3 separate wells of staining for SYTL4 with ab110519, 42 hours after application of either non-targeting siRNAs (top row) or pooled siRNAs targeting mouse sytl4 mRNA. **Lower panel –** Comparison of mean intensity of the images in the panel above, showing a significant reduction in staining following RNA-interference of *sytl4* (P=0.0183, unpaired t-test). Images from control well with cells and secondary antibody alone had a mean intensity of approximately 1500.

Supplementary methods III: Western Blot using antibody for mouse SYTL4 (abcam ab110519,RRID:AB_10858160)

Samples of SCN tissue were micro-dissected under a stereomicroscope from coronal brain slices, isolated using a brain matrix (Zivic Instruments,PA, USA) and skin graft blades (Swann-Morton Ltd., UK). The SCN tissue from three male C57BL/6J mice (all 11-12 weeks of age) at either ZT17-18 or ZT5-6 was pooled and compared to the pooled remains of the slices from the same three mice. All samples were snap frozen on dry ice and stored at -80°C until processing. All samples were homogenized in RIPA buffer with Protease inhibitors (Sigma Aldritch Ltd., UK) and the centrifuged at 16,00x g at 4°C for 10min. The supernatant was mixed 1:1 with x2 laemmli buffer (Sigma Aldritch Ltd., UK) and protein concentrations assessed using comparison to a protein standard curve (micro BCA protein assay kit, Thermo Fisher Scientific Inc., UK). 30µg of each sample was run on a BioRad Criterion Precast gel (4-20%, Bio-Rad Laboratories Ltd., UK), then transferred to a 0.2µm PVDF membrane using a BioRad Trans-Blot Turbo (Bio-Rad Laboratories Ltd., UK). Blocking and antibody labelling was carried out in Tris-buffered saline with 0.1% v/v Tween20 (TBS-T) and 5% milk powder (w/v). A 2hr block at 20^{0}C was followed by followed by staining with primary antibody for SYTL4 (1:500) rabbit polyclonal, ab110519, Abcam plc, Cambridge, UK, RRID:AB_10858160). The secondary antibody was Donkey anti-rabbit HRP (ab98503, Abcam plc, Cambridge, UK), applied for 1.5hrs at 20^{0}C. HRP-staining was revealed using Luminata Forte HRP substrate (Millipore, UK) for 5min and the bands visualised using an X-ray film-developer (Xograph Healthcare).All images were analysed for densitometry using Li-Cor image studio lite (version 5.2, LI-COR, Inc.).

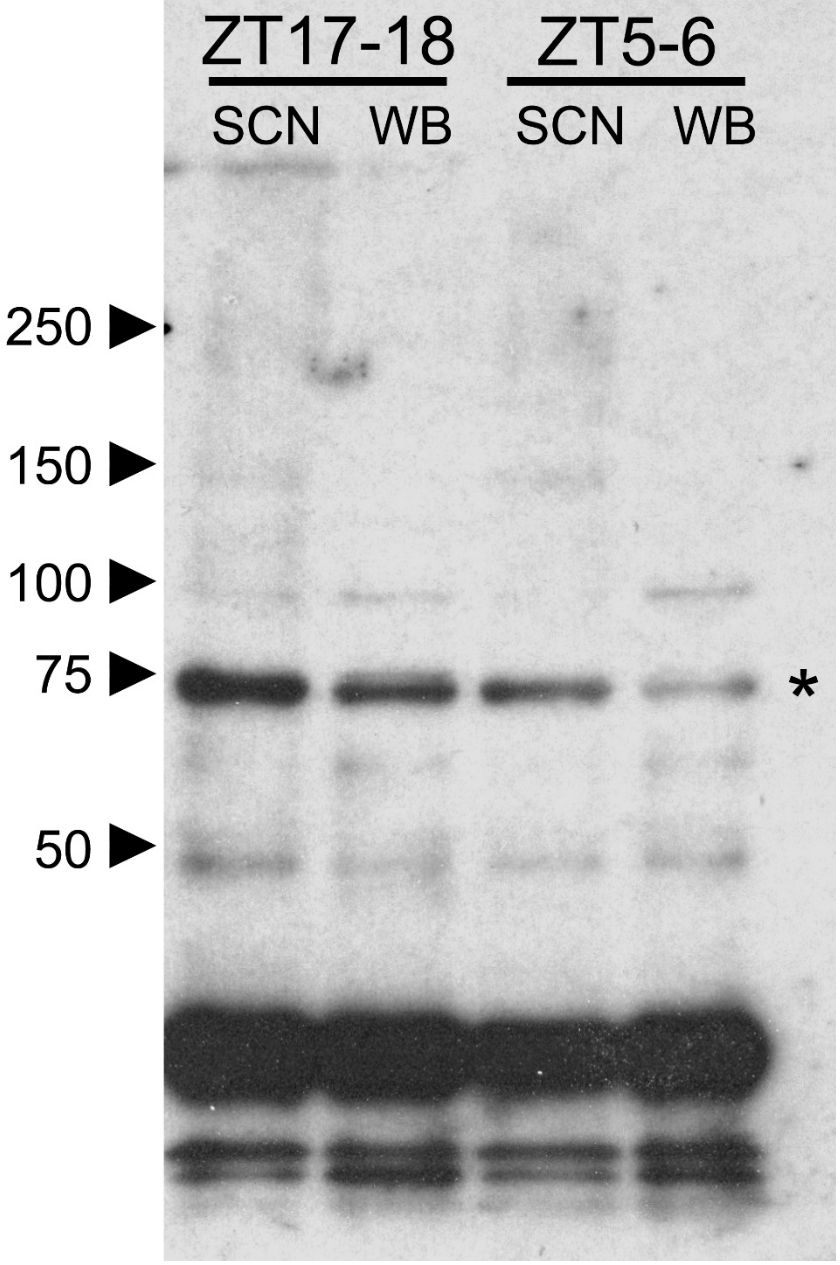

Figure: Left – Western blot for SYTL4 with ab110519, all lanes loaded with 30µg of protein.

Densitometry of the bands around 76 kDa in size (*, isoform1, http://www.uniprot.org/uniprot/Q9R0Q1) indicates a 1.53-fold enrichment for SYLT4 in the SCN compared to surrounding brain tissue at ZT17-18 and a 2.44-fold enrichment for SYLT4 in the SCN compared to surrounding brain tissue at ZT5-6.

Supplementary files:

Supplementary _file1: Extended Table 1 and Supplementary tables (.pdf) -

Extended Table 1	A more detailed version of table 1, with additional metadata on the resources used for the studies that comprise the meta-analysis
Supplementary Table 1	The overlap between identifiers for different Affymetrix microarray platforms and RNA-Seq data.
Supplementary Table 2	The significant (q<0.01) depletion of transcripts for a number of immediate early genes (IEGs) in the SCN compared to the whole brain.
Supplementary Table 3	The overlap between the 4403 MGI gene symbols for enriched and depleted transcripts in the SCN (at 1% FDR) and the 45 known imprinted genes presented in Figure1 of Gregg, et al.2010 Science 329.doi:10.1126/science.1190830. Values in bold are those with M* >3.

Supplementary _file2 (HTML file, .html) - A interactive version of figure 2, providing the ability to zoom in on individual gene symbols. Hover over points to see values for combined effect size (M*), q-value and I-squared values.

Supplementary _file3 (spreadsheet, .csv) - Full table of all results of the meta-analysis,

Column Key

MGI symbol	Mouse Gene Information official symbol
Ensembl Gene ID	Corresponding ENSEMBL Mouse Gene ID
Description	Gene Description
For each transcriptomic platform:<br>Enrich<br>Hedges_g<br>Var_g	 Fold Enrichment for platform (SCN vs Whole Brain, Log2)<br>Effect Size based on Fold<br>Variance of Effect Size
df	Degrees of freedom (studies for which there was data -1)
I^2	I-squared value (amount of variation that is not explained by variation within studies)
Tau^2	Variation between studies
REM_M*	Combined Effect Size for the meta-analysis
REM_pVal	p-value for the meta-analysis
q-value	pFDR-adjusted p-value for the meta-analysis

Supplementary _file4 (Excel spreadsheet, .xls) - Full Output from BiNGO analysis of overrepresented GO terms, for 426 enriched gene symbols and 611 depleted gene symbols. Each tab is a table for Ontological terms for either Biological Process, Cell Component, or Molecular Function.

Extended Table1

accession code	file code	SCN or WB	Age	Strain	Sex	Array type	PMID	circadian time
GSE6904	GSM159292	SCN	adult	C57 BL/6	male	430A_v2	18021443	ZT15-16
GSE6904	GSM159293	SCN	adult	C57 BL/6	male	430A_v2	18021443	ZT15-16
GSE6904	GSM159294	SCN	adult	C57 BL/6	male	430A_v2	18021443	ZT15-16
GSE7814	GSM189596	WB	10-12 weeks	C57 BL/6	male	430A_v2	17991715	?
GSE7814	GSM189598	WB	10-12 weeks	C57 BL/6	male	430A_v2	17991715	?
GSE7814	GSM189600	WB	10-12 weeks	C57 BL/6	male	430A_v2	17991715	?
GSE7814	GSM189602	WB	10-12 weeks	C57 BL/6	male	430A_v2	17991715	?
GSE7814	GSM189628	WB	10-12 weeks	BALB/c	male	430A_v2	17991715	?
GSE7814	GSM189630	WB	10-12 weeks	BALB/c	male	430A_v2	17991715	?
GSE7814	GSM189632	WB	10-12 weeks	BALB/c	male	430A_v2	17991715	?
GSE7814	GSM189634	WB	10-12 weeks	BALB/c	male	430A_v2	17991715	?
GSE16496	GSM414571	SCN	7-8 weeks	BALB/c	male	430_v2	21858037	pooled ZT0, 4, 8, 12, 16, 20
GSE16496	GSM414572	SCN	7-8 weeks	BALB/c	male	430_v2	21858037	pooled ZT0, 4, 8, 12, 16, 21
GSE28574	GSM707557	SCN	2 months	C57 BL/6	male	430_v2	21610730	pooled CT2 and CT14
GSE20411	GSM511616	WB	8 weeks	C57 BL/6	male/ female	430_v2	20526689	?
GSE20411	GSM511617	WB	8 weeks	C57 BL/6	male/ female	430_v2	20526689	?
GSE20411	GSM511618	WB	8 weeks	C57 BL/6	male/ female	430_v2	20526689	?
GSE20411	GSM511619	WB	8 weeks	C57 BL/6	male/ female	430_v2	20526689	?
GSE20411	GSM511620	WB	8 weeks	C57 BL/6	male/ female	430_v2	20526689	?
GSE20411	GSM511621	WB	2yrs	C57 BL/6	male/ female	430_v2	20526689	?
GSE20411	GSM511622	WB	2yrs	C57 BL/6	male/ female	430_v2	20526689	?
GSE20411	GSM511623	WB	2yrs	C57 BL/6	male/ female	430_v2	20526689	?
GSE20411	GSM511624	WB	2yrs	C57 BL/6	male/ female	430_v2	20526689	?
GSE20411	GSM511625	WB	2yrs	C57 BL/6	male/ female	430_v2	20526689	?
GSE9954	GSM252077	WB	10-12 weeks	C57 BL/6	male	430_v2	18365009	?
GSE9954	GSM252078	WB	10-12 weeks	C57 BL/6	male	430_v2	18365009	?
GSE9954	GSM252079	WB	10-12 weeks	C57 BL/6	male	430_v2	18365009	?
MEXP-3933	WT_sham_1	SCN	40 days +	OPN4wt	male	MoEx 1.0 ST	23993098	CT16
MEXP-3933	WT_sham_2	SCN	40 days +	OPN4wt	male	MoEx 1.0 ST	23993098	CT16
MEXP-3933	WT_sham_3	SCN	40 days +	OPN4wt	male	MoEx 1.0 ST	23993098	CT16
MEXP-3933	WT_sham_4	SCN	40 days +	OPN4wt	male	MoEx 1.0 ST	23993098	CT16
MEXP-3933	WT_sham_5	SCN	40 days +	OPN4wt	male	MoEx 1.0 ST	23993098	CT16
MEXP-3933	WT_sham_6	SCN	40 days +	OPN4wt	male	MoEx 1.0 ST	23993098	CT16
GSE27282	GSM674605	WB	adult	C57 BL/6	male	MoEx 1.0 ST	21625610	
GSE27282	GSM674606	WB	adult	C57 BL/6	male	MoEx 1.0 ST	21625610	
GSE27282	GSM674607	WB	adult	C57 BL/6	male	MoEx 1.0 ST	21625610	
GSE27282	GSM674608	WB	adult	C57 BL/6	male	MoEx 1.0 ST	21625610	
GSE27282	GSM674609	WB	adult	C57 BL/6	male	MoEx 1.0 ST	21625610	

GSE27282	GSM674610	WB	adult	C57 BL/6	male	MoEx 1.0 ST	21625610	
GSE27282	GSM674611	WB	adult	C57 BL/6	male	MoEx 1.0 ST	21625610	
GSE27282	GSM674612	WB	adult	C57 BL/6	male	MoEx 1.0 ST	21625610	
GSE27282	GSM674613	WB	adult	C57 BL/6	male	MoEx 1.0 ST	21625610	
GSE27282	GSM674614	WB	adult	C57 BL/6	male	MoEx 1.0 ST	21625610	
GSE27282	GSM674615	WB	adult	DBA/2	male	MoEx 1.0 ST	21625610	
GSE27282	GSM674616	WB	adult	DBA/2	male	MoEx 1.0 ST	21625610	
GSE27282	GSM674617	WB	adult	DBA/2	male	MoEx 1.0 ST	21625610	
GSE27282	GSM674618	WB	adult	DBA/2	male	MoEx 1.0 ST	21625610	
GSE27282	GSM674619	WB	adult	DBA/2	male	MoEx 1.0 ST	21625610	
GSE27282	GSM674620	WB	adult	DBA/2	male	MoEx 1.0 ST	21625610	
GSE27282	GSM674621	WB	adult	DBA/2	male	MoEx 1.0 ST	21625610	
GSE27282	GSM674622	WB	adult	DBA/2	male	MoEx 1.0 ST	21625610	
GSE27282	GSM674623	WB	adult	DBA/2	male	MoEx 1.0 ST	21625610	
GSE27282	GSM674624	WB	adult	DBA/2	male	MoEx 1.0 ST	21625610	
GSE27282	GSM674625	WB	adult	DBA/2	male	MoEx 1.0 ST	21625610	
GSE27282	GSM674626	WB	adult	DBA/2	male	MoEx 1.0 ST	21625610	
E-MEXP-3493	WT_ZT6_1	SCN	3 months	C3H/HeJ	male	MoGene 1.0 ST	22264613	ZT6
E-MEXP-3493	WT_ZT6_2	SCN	3 months	C3H/HeJ	male	MoGene 1.0 ST	22264613	ZT6
E-MEXP-3493	WT_ZT6_3	SCN	3 months	C3H/HeJ	male	MoGene 1.0 ST	22264613	ZT6
E-MEXP-3493	WT_ZT6_4	SCN	3 months	C3H/HeJ	male	MoGene 1.0 ST	22264613	ZT6
E-MEXP-3493	WT_ZT11_1	SCN	3 months	C3H/HeJ	male	MoGene 1.0 ST	22264613	ZT11
E-MEXP-3493	WT_ZT11_2	SCN	3 months	C3H/HeJ	male	MoGene 1.0 ST	22264613	ZT11
E-MEXP-3493	WT_ZT11_3	SCN	3 months	C3H/HeJ	male	MoGene 1.0 ST	22264613	ZT11
E-MEXP-3493	WT_ZT11_4	SCN	3 months	C3H/HeJ	male	MoGene 1.0 ST	22264613	ZT11
E-MEXP-3493	WT_ZT13_1	SCN	3 months	C3H/HeJ	male	MoGene 1.0 ST	22264613	ZT13
E-MEXP-3493	WT_ZT13_2	SCN	3 months	C3H/HeJ	male	MoGene 1.0 ST	22264613	ZT13
E-MEXP-3493	WT_ZT13_3	SCN	3 months	C3H/HeJ	male	MoGene 1.0 ST	22264613	ZT13
E-MEXP-3493	WT_ZT13_4	SCN	3 months	C3H/HeJ	male	MoGene 1.0 ST	22264613	ZT13
GSE34469	GSM849761	WB	70 days	C57 BL/6	male	MoGene 1.0 ST	23580197	
GSE34469	GSM849762	WB	70 days	C57 BL/6	male	MoGene 1.0 ST	23580197	
GSE34469	GSM933084	WB	70 days	C57 BL/6	male	MoGene 1.0 ST	23580197	
GSE34469	GSM933085	WB	70 days	C57 BL/6	male	MoGene 1.0 ST	23580197	
GSE34305	GSM847026	WB	70 days	C57 BL/6	male	MoGene 1.0 ST	22560501	
GSE34305	GSM847027	WB	70 days	C57 BL/6	male	MoGene 1.0 ST	22560501	
GSE34305	GSM847029	WB	70 days	C57 BL/6	male	MoGene 1.0 ST	22560501	
GSE34305	GSM847031	WB	70 days	C57 BL/6	male	MoGene 1.0 ST	22560501	
GSE34305	GSM847033	WB	70 days	C57 BL/6	male	MoGene 1.0 ST	22560501	
GSE24940	GSM613009	WB	10-12 weeks	C57 BL/6	male	MoGene 1.0 ST	21088282	
GSE24940	GSM613010	WB	10-12 weeks	C57 BL/6	male	MoGene 1.0 ST	21088282	
GSE24940	GSM613011	WB	10-12 weeks	C57 BL/6	male	MoGene 1.0 ST	21088282	

PRJEB9284	SAMEA3368226	SCN	adult	C3H/HeH x C57 BL/6J	male	RNA-Seq	26232227	ZT3
PRJEB9284	SAMEA3368227	SCN	adult	C3H/HeH x C57 BL/6J	male	RNA-Seq	26232227	ZT3
PRJEB9284	SAMEA3368228	SCN	adult	C3H/HeH x C57 BL/6J	male	RNA-Seq	26232227	ZT3
PRJEB9284	SAMEA3368229	SCN	adult	C3H/HeH x C57 BL/6J	male	RNA-Seq	26232227	ZT15
PRJEB9284	SAMEA3368230	SCN	adult	C3H/HeH x C57 BL/6J	male	RNA-Seq	26232227	ZT15
PRJEB9284	SAMEA3368237	SCN	adult	C3H/HeH x C57 BL/6J	male	RNA-Seq	26232227	ZT15
PRJNA235222	SAMN02585109	SCN	11-12 weeks	C57 BL/6	male	RNA-Seq	24531307	CT4 , NT cycle
GSE43013	GSM1055111	WB	adult	C57 BL/6	male	RNA-Seq	25677554	
GSE30352	GSM752614	WB	adult	C57 BL/6	female	RNA-Seq	22012392	
GSE30352	GSM752615	WB	adult	C57 BL/6	male	RNA-Seq	22012392	
PRJEB2494	SAMEA811980	WB	8 weeks	C57 BL/6	male	RNA-Seq	PMC3428933	
PRJEB2494	SAMEA811978	WB	8 weeks	C3H/HeJ	male	RNA-Seq	PMC3428933	
GSE41338	GSM1015150	WB	adult	C57 BL/6	pooled male & female	RNA-Seq	23258891	
GSE41338	GSM1015151	WB	adult	C57 BL/6	pooled male & female	RNA-Seq	23258891	
GSE41637	GSM1020640	WB	8 weeks	DBA/2J	male	RNA-Seq	23258890	
GSE41637	GSM1020649	WB	8 weeks	C57 BL/6	male	RNA-Seq	23258890	
GSE41637	GSM1020657	WB	8 weeks	CD1	male	RNA-Seq	23258890	

Supplementary Table 1: The overlap between identifiers for different Affymetrix microarray platforms and RNA-Seq data. Numbers are overlapping MGI gene symbols for each platform and shaded values are the total number of unique gene symbols with data for each platform. No platform provides full coverage and the later microarray platforms have greater coverage than earlier platforms.

	MoEx	MoGene	RNASeq	430V2	430Av2	
MoEx	28235	22091	19204	18414	13316	
MoGene		24410	17534	17983	13318	
RNASeq			20308	16529	12094	
430V2				19669	14200	
430Av2					14200	
						31684 total

Supplementary Table 2: The significant (q<0.01) depletion of transcripts for a number of immediate early genes (IEGs) in the SCN compared to the whole brain.

MGI symbol	Description	Effect size (M*)	pFDR q-value
Fosb	FBJ osteosarcoma oncogene B	-1.80	1.85E-10
Npas1	neuronal PAS domain protein 1	-2.04	4.36E-04
Egr4	early growth response 4	-2.18	4.44E-10
Nr4a3	nuclear receptor subfamily 4, group A, member 3	-2.78	1.26E-03
Npas2	neuronal PAS domain protein 2	-2.95	8.90E-03
Homer3	homer homolog 3 (Drosophila)	**-3.14**	1.73E-03
Homer2	homer homolog 2 (Drosophila)	**-3.18**	2.03E-04
Nr4a1	nuclear receptor subfamily 4, group A, member 1	**-3.32**	1.08E-04
Arc	activity regulated cytoskeletal-associated protein	**-3.58**	2.67E-06
Nr4a2	nuclear receptor subfamily 4, group A, member 2	**-4.17**	2.47E-09
Plk2	polo-like kinase 2	**-4.23**	1.25E-04
Egr1	early growth response 1	**-4.64**	7.01E-06
Homer1	homer homolog 1 (Drosophila)	**-4.84**	2.92E-05
Nrn1	neuritin 1	**-6.15**	1.30E-12

Supplementary Table 3: The overlap between the 4403 MGI gene symbols for enriched and depleted transcripts in the SCN (at 1% FDR) and the 45 known imprinted genes presented in Figure1 of Gregg, et al.2010 *Science 329.doi:*10.1126/science.1190830. Values in bold are those with M* >3.

Symbol	Effect size (M*)	pFDR q-value
Calcr	**8.61**	7.17E-12
Usp29	**7.68**	2.14E-18
Nap1l5	**6.38**	7.34E-07
Ndn	**5.94**	1.48E-16
Impact	**5.64**	3.32E-04
Asb4	**5.64**	2.80E-29
Peg3	**4.97**	3.04E-05
Dlk1	**4.58**	1.27E-07
Peg10	**4.52**	2.86E-06
Zim1	**4.49**	2.00E-04
Grb10	**4.32**	1.32E-05
Ddc	**3.13**	8.09E-13
Slc22a3	1.99	8.60E-03
