# Supplementary material for: Meta-analysis of transcriptomic datasets identifies genes enriched in the mammalian circadian pacemaker
Source: Nucleic Acids Res. 2017 Aug 18;45(17):9860–73. doi: 10.1093/nar/gkx714 (PMC5737434; doi:10.1093/nar/gkx714)
Supplement: Supplementary Data [file gkx714_supp.zip › nar-00706-n-2017-File008.html]

cross\_select
